# Poly(*N*-isopropylacrylamide) and Copolymers: A Review on Recent Progresses in Biomedical Applications

**DOI:** 10.3390/gels3040036

**Published:** 2017-10-04

**Authors:** Sonia Lanzalaco, Elaine Armelin

**Affiliations:** 1 Industrial and Digital Innovation Department (DIID), Chemical Engineering, University of Palermo, Viale delle Scienze, Ed. 8, 90128 Palermo, Italy; sonia.lanzalaco@unipa.it; 2 Departament d’Enginyeria Química, EEBE, Universitat Politècnica de Catalunya, C/d’Eduard Maristany, 10-14, Building I, E-08019 Barcelona, Spain; 3 Barcelona Research Center in Multiscale Science and Engineering, Universitat Politècnica de Catalunya, Campus Diagonal Besòs (EEBE), C/d’Eduard Maristany 10-14, Edifici IS, 08019 Barcelona, Spain

**Keywords:** poly(*N*-isopropylacrylamide), thermo-responsive polymer, copolymers, biocompatibility, biodegradability, 4D-printing

## Abstract

The innate ability of poly(*N*-isopropylacrylamide) (PNIPAAm) thermo-responsive hydrogel to copolymerize and to graft synthetic polymers and biomolecules, in conjunction with the highly controlled methods of radical polymerization which are now available, have expedited the widespread number of papers published in the last decade—especially in the biomedical field. Therefore, PNIPAAm-based hydrogels are extensively investigated for applications on the controlled delivery of active molecules, in self-healing materials, tissue engineering, regenerative medicine, or in the smart encapsulation of cells. The most promising polymers for biodegradability enhancement of PNIPAAm hydrogels are probably poly(ethylene glycol) (PEG) and/or poly(ε-caprolactone) (PCL), whereas the biocompatibility is mostly achieved with biopolymers. Ultimately, advances in three-dimensional bioprinting technology would contribute to the design of new devices and medical tools with thermal stimuli response needs, fabricated with PNIPAAm hydrogels.

## 1. Introduction

Hydrogels are a class of three-dimensional macromolecular polymer network obtained from physical and/or chemical crosslinking which have high hydrophilicity but are insoluble in water [1]. Stimuli-responsive hydrogels are polymers that can undergo a tuneable sol–gel transition in response to external stimuli [2], such as pH [3,4,5], temperature [6,7], light [6,8], ionic strength [9,10], magnetic field [11,12], and/or the supply of electric field [13,14]. Among them, poly(*N*-isopropylacrylamide) (PNIPAAm) [15,16,17] is the most studied thermally actuating hydrogel, with thermo-reversible gelation properties in aqueous solutions, gelling at temperatures in the range of 32–35 °C and turning into a solution upon cooling. The reversibility of the hydrophilic/hydrophobic state occurs by varying the temperature below or above a critical value, well known as the lower critical solution temperature (LCST).

**Figure 1:**
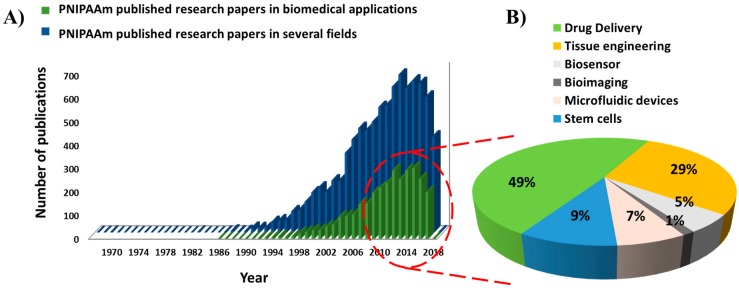
(**A**) Evolution of published research articles about poly(*N*-isopropylacrylamide) (PNIPAAm) and its application in biomedical and other fields, per year; and (**B**) Percentage of PNIPAAm published research articles in biomedical field, classified by application. Database used for the bibliographic analysis: Scopus^®^ (Elsevier, Amsterdam, The Netherlands).

PNIPAAm is particularly and extensively investigated due to its solubility in water and its LCST close to body temperature (around 36.5–37.5 °C) [18]. The hydrogel is easily accessible by radical polymerization to obtain variable architectures such as block copolymers [19,20,21,22,23], gels [24,25], or grafted polymers [26,27]. Since it was first reported in 1968 [28], several papers and reviews related to the employment of PNIPAAm in the field of biomedicine have been published. Figure 1A presents the evolution of the number of articles published every year on PNIPAAm hydrogels with applications in several fields, such as physics and astronomy, environmental science and energy, and particularly in the biomedical field. Among biomedical applications, the most explored areas are drug delivery (49% of the research articles) and tissue engineering (29%), followed by stem cells (9%), microfluidic devices (7%), biosensors (5%), and bioimaging (1%) (Figure 1B).

In summary, from Figure 1 it is possible to realize that over 8900 publications were published on PNIPAAm from 1967, 3190 of them being related to biomedical applications. Even if the first articles were published in 1986, an exponential interest in this thermo-responsive hydrogel has been detected in the last decade and continuous growth is envisaged.

The main innovations arising from the synthesis and the use of this polymer in biomedical applications are listed in Table 1.

Prompted by the noteworthy interest registered in the last decade, along with the high versatility of this polymer in biotechnological field, this review compiles and discusses selected highlighted papers of this period.

Therefore, the first part introduces the fundamental properties of PNIPAAm as a thermo-sensitive polymer, with the PNIPAAm phase transition’s dependence on the solvent nature and on the hydrogen-bond interactions. The second aspect mainly focuses on the biocompatibility and biodegradability of PNIPAAm hydrogel. After that, the recent development of copolymers and graft polymers with strong effect on the LCST of PNIPAAm will be detailed. Finally, the challenges and future prospects of responsive PNIPAAm molecules with controllable properties on the newest bioprinting and biointerface emerging areas were examined.

## 2. The Role of the Hydrogen-Bonding Interactions

**Figure 2:**
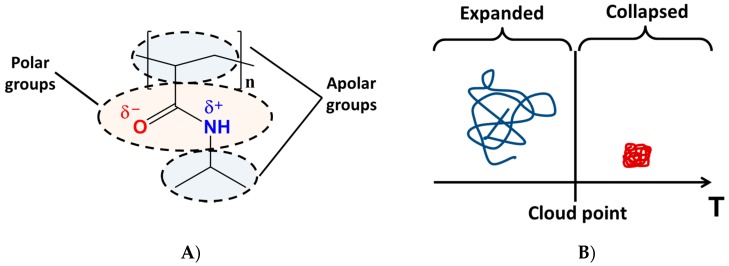
(**A**) Chemical formula of PNIPAAm and (**B**) Representation of volume phase transition between coil (**left**) and globular (**right**) hydrogel conformations.

At a cloud point, PNIPAAm exhibits a unique volume phase transition from a hydrated state called a hydrophilic state with expanded structure to a shrunken dehydrated state called a collapsed structure (Figure 2) [54,55,56]. The presence of hydrophilic and hydrophobic groups inside the neutral polymer is responsible for this reversible sol–gel behaviour of PNIPAAm homopolymer in water solutions. The reversibility of the hydrophilic/hydrophobic states occurs by varying the temperature below or above the LCST value (32 °C) [57]. The LCST is the temperature above which the gel becomes insoluble in an aqueous environment. LCST depends on the critical gel concentration (CGC). Then, at its CGC, solvated PNIPAAm molecules will exhibit aqueous insolubility upon heating above the LCST. The LCST is mainly dependent on the hydrogen bonding between water molecules and the structure of functional monomer units of PNIPAAm polymer; i.e., N–H and C=O linkages [58,59,60,61,62]. Thus, the incorporation of hydrophilic units typically increases the volume-phase transition temperature (VPTT), whereas the addition of hydrophobic units has the opposite effect.

The importance of the intermolecular force governed between highly electronegative atoms (i.e., N, O, F, and Cl) and hydrogen atoms—particularly in small organic molecules, inorganic acids, or among water molecules—is well known [63]. When a polymer is dissolved in water, there are three types of interactions that can take place: between polymer molecules, polymer chains, and water molecules, and solely between water molecules.

**Figure 3:**
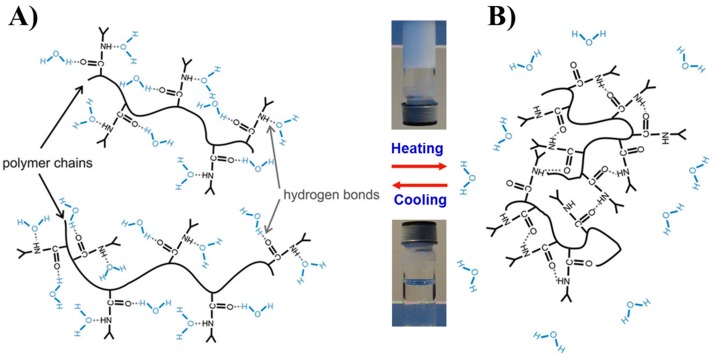
Representation of (**A**) the swollen PNIPAAm hydrosol in aqueous solution below *T*_c_ (32 °C) and (**B**) the shrunken dehydrated PNIPAAm hydrogel above *T*_c_ (32 °C). Adapted with permission from Reference [64]. Copyright ^©^ 2015 Springer Science & Business Media Singapore.

Besides the hydrogen-bonding, intermolecular and intramolecular interactions on PNIPAAm solutions are sensitive not only to temperature change but also to the nature of the solvent. Accordingly, two types of interactions between solvents and PNIPAAm molecules can be distinguished: (i) pure water and (ii) mixtures of water/organic solvents. Aqueous solutions of PNIPAAm become abruptly turbid at the cloud point (32 °C), easily switching to the liquid state when the temperatures decreases below 32 °C (Figure 3, inset photographs). When PNIPAAm changes its state from liquid to almost solid, a stabilization of the collapsed state is achieved. The scheme represented in Figure 3 illustrates this behaviour according to the number and type of hydrogen-bonding interactions upon switchable response [62].

**Figure 4:**
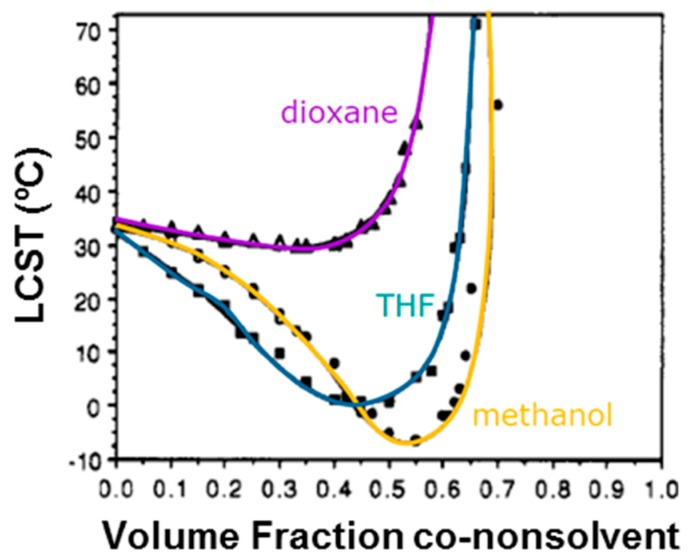
LCSTs of PNIPAAm (0.40 mg/mL) in water-*co*-nonsolvent mixtures. Adapted with permission from Reference [69]. Copyright © 1991 American Chemical Society.

**Figure 5:**
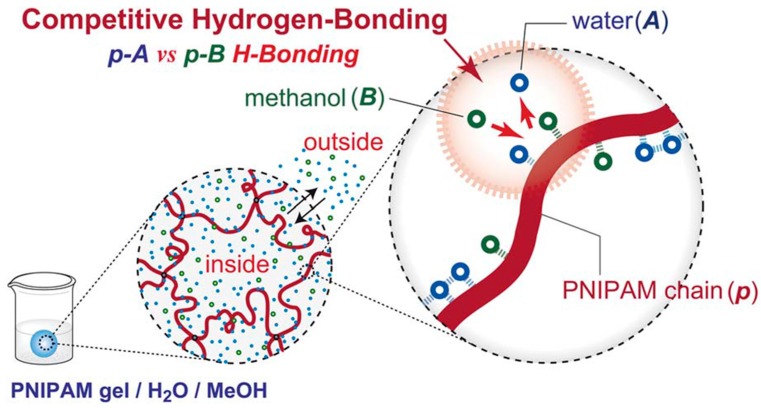
Model of competitive hydrogen-bond formation between polymer–water (*p*–*A*) and polymer–methanol (*p*–*B*) of PNIPAAm in both mixed solvents, proposed by Kojima and Tanaka. Reprinted with permission from Reference [67]. Copyright ^©^ 2012 The Royal Society of Chemistry.

To get a closer insight into the hydrogen bonding interactions in mixed water/organic solvents of PNIPAAm hydrogel, several works have been published and different mechanisms have been proposed [64,65,66,67]. The second solvent is called co-nonsolvent. As examples of good co-nonsolvents, dioxane, tetrahydrofuran (THF), or methanol are commonly explored [68,69]. Tirrell and co-workers [69] observed that methanol and water are excellent co-nonsolvents mixtures for poly(*N*-isopropylacrylamide). The addition of MeOH provokes a depression of the cloud point and a sudden increase of the gel solubility. This phenomenon is also observed with dioxane and THF as second solvents (Figure 4). More recently, Kojima and Tanaka [67] reported a detailed thermodynamic study where they explicitly demonstrated—using theoretical calculations and experimental data—the molecular mechanism of co-nonsolvency in PNIPAAm hydrogel in mixtures of water/methanol. It is governed by the cooperative dehydration caused by the decreasing of the water composition and the blocking of the hydrogen-bonding sites by the formation of new polymer–methanol hydrogen-bonds. Therefore, the hydration and dehydration are cooperative phenomena. They named this theory the re-entrant volume phase transition of crosslinked gels. Figure 5 illustrates their competitive model for co-nonsolvency.

Thus, the chain conformation depends strongly on fraction of co-nonsolvent. Moreover, depending on what a PNIPAAm gel interacts with, it will have different behaviours and cloud point. Organogels are a clear example on how the several types of weak forces among macromolecular chains affect not only the formation of polymer aggregates, but also the structure and physical properties of the gel. The fundamental principles that govern the swelling equilibrium and dynamics also depend on the gelling agent (gelator), which itself can be of several types, offering transient or permanent bonding. When using chemical crosslinkers, the bridging molecules create two- and three-dimensional aggregates. Tanaka and co-workers [70] proved that the volume phase transition in chemically crosslinked gels is based on the contribution of both the hydrogen-bonding and the van der Waals interactions between molecules.

Several other parameters can influence the volume-phase transition temperature (VPTT) of PNIPAAm hydrogel, as such as the molecular weight, presence of end groups, tacticity, and especially the presence of hydrophilic or hydrophobic comonomers in PNIPAAm copolymers and graft polymers. More detailed discussion on how such parameters will affect the PNIPAAm hydrogel properties can be found in several reviews recently published on thermo-responsive hydrogels [1,62].

## 3. Biocompatibility and Biodegradability of PNIPAAm-Based Hydrogels

Biodegradability of polymers is affected by both their chemical and physical properties (melting point, glass transition temperature, crystallinity, storage modulus, etc.) [71]. Poor biodegradability of PNIPAAm hydrogel has limited its application in clinical medicine. Several strategies for the preparation of biodegradable PNIPAAm-based hydrogels are being explored by the introduction of different biodegradable monomers and/or crosslinkers or natural polymers, such as poly(amino acids) [72], polysaccharides [73,74], proteins [27], and synthetic polymers like poly(esters) [75,76], poly(caprolactone) [77,78], and poly(ethylene glycol) (PEG) [79,80]. Depending on the application target, biodegradability and biocompatibility are both required properties (e.g., in drug delivery and cell encapsulation).

**Figure 6:**
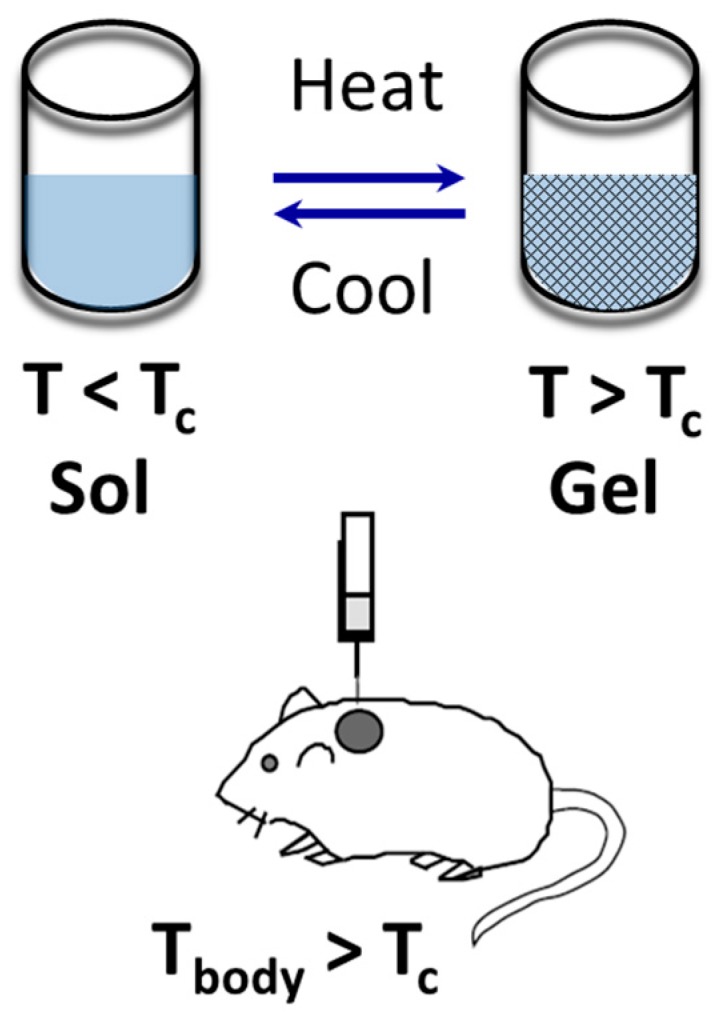
Sketch of subcutaneous injection of drug-delivery hydrogel containing bioactive molecules, with reversible sol–gel transition around the LCST point. *T*_c_ is the coil–globular critical temperature of a LCST hydrogel. Adapted with permission from reference [81]. Copyright © 1997 Nature Macmillan Publishers Ltd. (Basingstoke, UK) 1997.

In drug delivery systems, the hydrogel can be loaded with bioactive molecules in an aqueous phase at moderate temperature (around 32 °C), where they form a sol. In this physical state, the polymer is injectable. Upon subcutaneous injection and subsequent rapid heating to the body temperature (around 36.5–37.5 °C), the loaded polymer forms a gel that can act as a sustained-release matrix for drugs. The bioactive molecules entrapped in the polymer gel will be released in the body at first by diffusion, and later by the combination of both diffusion and degradation mechanisms; i.e., if the hydrogel is biodegradable. Figure 6 illustrates the idea used by Jeong et al. [81] for injectable hydrogels.

Polymer degradation also influences the release kinetics. In some systems (e.g., wound dressing and surgical sutures for abdominal hernia repair), polymer degradability is not desirable. In these materials, the efficiency of antibiotic or antibacterial release is a very important parameter for the healing process or to avoid inflammatory reactions in the host tissue, and biocompatibility is obviously mandatory. In drug delivery systems, both properties (i.e., biodegradability and biocompatibility [82]) are desirable, or the drug carrier should at least be removed from the body by other routes. Thus, in the design of PNIPAAm copolymers and graft polymers, these properties should be modulated according to the specific application purpose.

**Figure 7:**
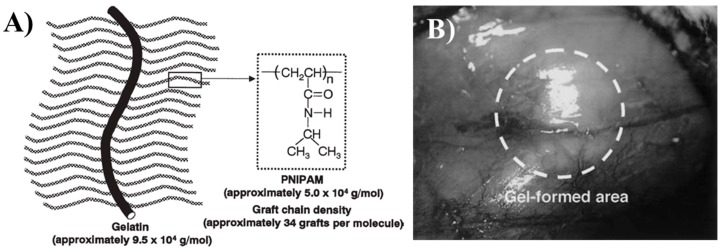
(**A**) Representation of the structure of poly(*N*-isopropylacrylamide)-grafted gelatin (PNIPAAm–gelatin) and (**B**) PNIPAAm–gelatin gel formation in rat subcutaneous tissue. Reprinted from reference [83]. Copyright © 2004 The Japanese Society for Artificial Organs.

PNIPAAm is highly biocompatible with animal cells. Matsuda and co-workers [83] reported the in situ PNIPAAm–gelatin gel formation in the subcutaneous tissue of a rat (Figure 7). They histologically examined—after organogel injection—the rat fibroblasts formation for up to 12 weeks. The inflammation reaction appeared at the initial stage of injection, but subsided in two weeks and the fibroblasts inside the gel extended and proliferated. This work proves the feasibility of PNIPAAm–gelatin gel implantation as cell scaffold in vivo. In a similar way, other studies for in vivo tests with PNIPAAm hydrogel were reported. For example, water-soluble chitosan-*g*-PNIPAAm gel was injected in the submucosal layer of the bladder of rabbits, and good cartilage formation was obtained after 14 weeks [84]. Copolymers of PNIPAAm-chitosan were also tested for ocular drug delivery [85]. The copolymer was used to encapsulate timolol maleate molecules with a strong capacity to reduce the intra-ocular pressure (IOP), over a period of 12 h. Once again, the non-cytotoxicity of PNIPAAm–chitosan applied in vivo was observed, opening new insights for the treatment of glaucoma and other eye diseases employing this thermo-sensitive hydrogel.

**Figure 8:**
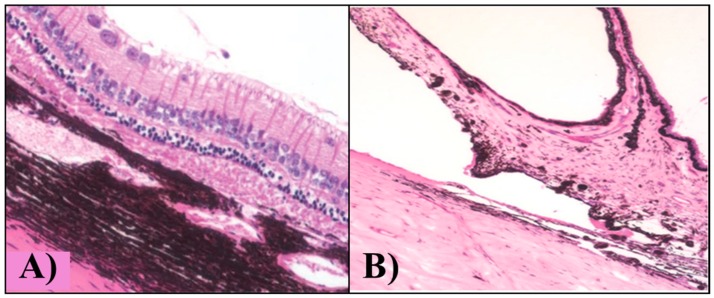
Light microscopy of the rabbit eye (**A**) retina and (**B**) anterior chamber after 6 months of intravitreal injection of PNIPAAm hydrogel. Ganglion cell layer and iris tissue are both facing upwards in (A) and (B). Reprinted with permission from reference [49]. Copyright © 2016 Hindawi Publishing Corporation (Cairo, Egypt).

Another very recent study [49] revealed ocular biocompatibility of pure PNIPAAm intravitreally injected in rabbit eyes (Figure 8). The moderate levels of inflammatory reaction in the conjunctiva—generally observed at the first stages—completely disappeared after the third month of implantation, proving that PNIPAAm hydrogel is nontoxic and are safe for in vivo applications in rabbits. They affirmed the iris and the anterior chamber angle showed no abnormalities or damage.

**Figure 9:**
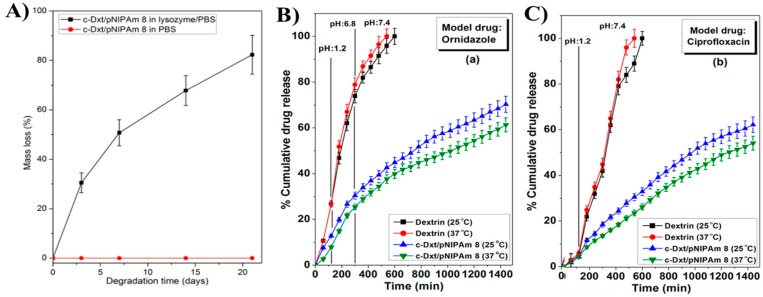
(**A**) Progressive mass loss of c-Dxt/PNIPAAm 8 xerogel films (■) in lysozyme/PBS and (●) in PBS only; (**B**) IOn vitro release of ornidazole from dextrin and c-Dxt/PNIPAAm 8; (**C**) In vitro release of ciprofloxacin from dextrin and c-Dxt/PNIPAAm 8. “Dxt” refers to dextrin biopolymer, whereas the letter “c” refers to covalently cross-linked hydrogel and the number 8 is related to the molar ratio described on Table 1 inside the article. Results represented are mean ± SD, *n* = 3. Reprinted with permission from reference [86]. Copyright © 2015 American Chemical Society.

Fortunately, by tuning the molecular structure of PNIPAAm with synthetic degradable and/or biopolymer materials, biodegradability and biocompatibility can be easily achieved. Das et al. [86] successfully synthesized covalently crosslinked PNIPAAm hydrogel using dextrin as biopolymer, NIPAm as monomer, *N*,*N′*-methylenebisacrylamide (MBA) as crosslinker, and potassium persulfate (KPS) as initiator for oral drug delivery applications. The hydrogel showed excellent enzymatic degradation after incubation in lysozyme/phosphate-buffered saline (PBS) medium, reaching a mass loss of 80% in only 21 days (Figure 9A). Moreover, in vitro cytotoxicity and cell proliferation studies revealed a slightly enhanced DNA amount in synthesized hydrogel compared to the tissue culture plate (TCP) that was used as control. Additionally, the bio-organogel was able to deliver ornidazole and ciprofloxacin drugs in a controlled way (Figure 9B,C). The new PNIPAAm hydrogel is non-toxic, degradable in nature, and compatible for the controlled delivery of ornidazole and ciprofloxacin drugs. The pH- and temperature-responsive drug delivery studies were performed both in vitro and in vivo (albino rabbits).

To conclude, copolymerization of NIPAAm with a monomer which not only adjusts the LCST of PNIPAAm hydrogel but also introduces reactive functional groups and degradability is highly desired.

## 4. Poly(*N*-isopropylacrylamide) Copolymers and Graft Polymers

In 2007, a detailed review on PNIPAAm synthesis and copolymerization was reported by Rzaev et al. [87]. According to them, the most important synthesis routes for obtaining stimuli-responsive *N*-isopropylacrylamide copolymers are: (i) free radical polymerization (FRP); (ii) atom transfer radical polymerization (ATRP); and (iii) graft copolymerization—these polymerization methods are still used today. Generally, by conventional radical polymerization, monomers, initiators, and crosslinkers must have good solubility or dispersion in water. Free radical polymerization has several advantages, which include ease of controlling reaction conditions, suitability for a wide variety of monomers, and the employment of a non-toxic solvent (water). However, it has several disadvantages, including problems controlling particle size, degree of polymerization, polydispersity, and control of the polymer microstructure. Through the introduction of ATRP synthesis [88,89,90], hydrogels have been prepared by the simple addition of an initiator (usually an alkyl halide), a catalyst/activator (organometallic complexes), and the monomer and comonomer. Matyjaszewski and co-workers are ATRP pioneers, and developed several variants for the initiation of the ATRP reactions, such as initiators for continuous activator regeneration (ICAR); activator regenerated by electron transfer (ARGET); supplemental activator and reducing agent (SARA); and electrochemically-mediated ATRP (eATRP); among others [91,92,93]. These ATRP variants are more environmentally benign than the conventional one initially developed. All of them are controlled reaction polymerizations also employed for the preparation of homopolymers and copolymers of PNIPAAm, as well as for obtaining graft polymers. Each process has some advantages and limitations. The main advantages of the ATRP technique are the control of reaction kinetics and of the molecular weight, with relatively low dispersion and—most importantly—the possibility to obtain and to regulate complex molecular architectures, in terms of chain topology. Another very appealing study making the ATRP process very green is the “ATRPases”—a new ATRP methodology employing hemoprotein horseradish peroxidase (HRP) as catalyst for the polymerization of *N*-isopropylacrylamide [94,95]. Under ARGET ATRP conditions and in the presence of an alkyl bromide as initiator and sodium ascorbate as precursor of the reducing agent, bromine-terminated polymers with polydispersity indices (PDIs) as low as 1.44 were obtained. This work reported for the first time the use of the enzymatic catalyst in the absence of peroxides.

Different methods have been reported for graft copolymerization: (i) hydrogel copolymer grafted onto solid surfaces; (ii) photo-induced grafting copolymerization; (iii) plasma-induced grafting copolymerization; and (iv) irradiation copolymerization. We do not intend on describing each method in detail due to the extensive literature available [96,97].

By far, most of the swelling hydrogels are produced in bulk, solution, and emulsion polymerization. With respect to the medium of polymerization, solution and bulk are homogenous phases, while emulsion polymerization is conducted in heterogeneous phases. With the first two methods, the PNIPAAm hydrogels are usually obtained with random distribution of particle size, whereas with the latter, micro- and nano-sizes can be modulated. The monomer is usually dissolved in the dispersed phase, and a surfactant is dissolved in the organic phase to help the monomer and other aqueous reagents to be effectively dispersed throughout the continuous phase. Although particles with desirable sizes can be obtained by this technique, some disadvantages (e.g., the removal of the surfactants and organic solvents) are a very challenging problem [97].

### 4.1. Poly(N-isopropylacrylamide) Thermo-Responsive Hydrogels with Synthetic Polymers

**Figure 10:**
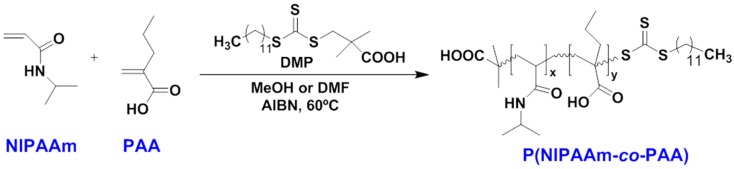
Scheme of P(NIPAAm-*co*-PAA) copolymer synthesis reported by Stayton and co-workers [98].

Copolymerization of NIPAAm with acrylic acid (AA) and acrylate monomers—such as 2-hydroxyethyl methacrylate (HEMA) and poly(ε-caprolactone) dimethacrylate (PCLDMA)—is widely used to develop biomaterials. Extensive research works have been published in the last few years. Stayton and co-workers [98] introduced the hydrophobic monomer butyl acrylate to tune the pH-responsive gelation behaviour of a copolymer composed by PNIPAAm and propylacrylic acid (PAA). They synthesized the copolymer by reversible addition fragmentation chain transfer (RAFT) polymerization (Figure 10), for gradual dissolution of drug with pH changes. RAFT is a reversible deactivation radical polymerization (RDRP) invented by Chiefari et al. in 1998 [99]. The RAFT mechanism, typically involving reagents as di-thioesters, is a living reaction in which transfer of the S=C(Z)S– moiety from a S=C(Z)S–R reagent (where R = free radical leaving group and Z = group controls C=S bond reactivity) between active and dormant chains serves to maintain the living character of the polymerization. The choice of Z and R in reagents is crucial to the success of the RAFT process. To ensure a high transfer constant, Z should activate (or at least not deactivate) the C=S double bond toward radical addition. The hydrogel dissolution and the vascular endothelial growth factor (VEGF) release profile were monitored, as well as the bioactivity using C2C12 mouse myoblasts—both in vitro. The copolymer with high PAA content (17 mol %) was able to maintain the bioactivity basic fibroblast growth factor (bFGF) following storage of hydrogel at 37 °C for 40 h. They also observed that changes in the molecular weight of P(NIPAAm-*co*-PAA) did not affect the gelation temperature for a given pH; instead, the increase of the hydrophobicity with comonomers like butyl acrylate (BA, employed to obtain P(NIPAAm-*co*-PAA-*co*-BA) triblock copolymer) favoured the formation of gel at higher pH, which does not occur with the diblock copolymer. Thus, the presence of acrylic acid units inside the polymer chains—which offer an amphiphilic character regulated by pH—would potentiate the dissolution and elimination of the non-biodegradable P(NIPAAm-*co*-PAA) copolymer. They propose that their system would be ideal for the delivery of angiogenic growth factors, when restoration of physiological pH is achieved with the subsequent tissue healing.

Most hydrophilic comonomers—as is the case of acrylics, including acrylic acid, acrylamide, and its derivatives—will offer not only higher biodegradability property, but also improved cell adhesion of the copolymer or graft polymer on life systems. For example, Matsunaga and co-workers recently [23] reported the enhanced cell adhesion of PNIPAAm hydrogel with a catechol moiety incorporated on 3-hydroxytyramine hydrochloride-derived dopamine methacrylamide (DMA) monomer. Dopamine (DA) is a well-known neurotransmitter involved in motor and cognitive functions. The modification of acrylate monomers with DA units helps to adjust the hydrophilic/hydrophobic balance of the overall polymer. In this study, they proved that DMA comonomer is able to enhance the initial attachment of PNIPAAm to cells and also to spread of cells on the organogel, thanks to the presence of the catechol groups and to the good mechanical strength generated by cohesive couplings. Additionally, their system did not show any cell detachment at temperatures lower than the LCST of PNIPAAm, corroborating the high gel stability of this system.

**Figure 11:**
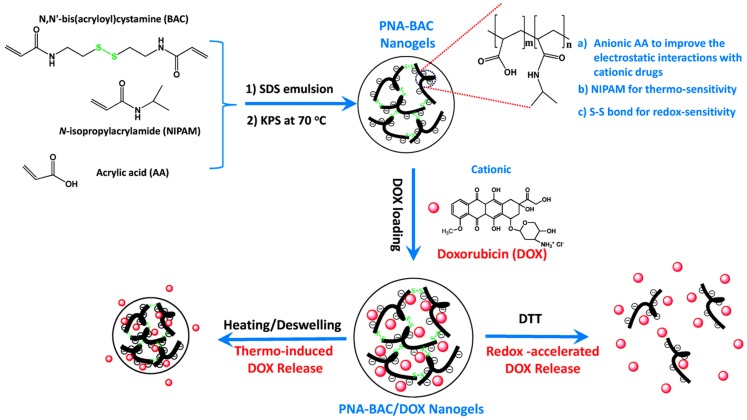
Scheme of preparation of PNA-BAC/DOX nanogels for in vitro anticancer drug release, reported by Zhan et al. [102]. Reprinted with permission from reference [102]. Copyright © 2015 The Royal Society of Chemistry.

Microgels and nanogels from NIPAAm and acrylic monomers have also been studied [100,101]. Zhan et al. [102] recently reported the synthesis of poly(*N*-isopropylacrylamide-*co*-acrylic acid) nanogels—named PNA-BAC NGs—for anticancer drug delivery. The copolymer was obtained by emulsion polymerization of NIPAAm, AA, and *N*,*N′*-bis(acryloyl)cystamine (BAC) acting as intracellular degradable crosslinker. Further, the NG was encapsulated with the cationic anticancer drug doxorubicin (DOX, Figure 11) to test the intracellular drug absorption and cytotoxicity. The employment of BAC comonomer is an interesting idea to improve the DOX intracellular uptake due to the ability of the cytoplasm, mitochondria, and nucleus of cells to cleave disulphide bonds. PNA-BAC NGs had higher encapsulation efficiency and release compared to the less-biodegradable comonomer (PNA-MBA NGs). In both copolymers, the LCST temperatures were 35.5 and 36.1 °C, respectively, for PNA-BAC NGs and PNA-MBA NGs, which are slightly higher than pure PNIPAAm. In conclusion, the combination of hydrophilic comonomers like AA and MBA, which render higher VPTT temperatures, with BAC molecules (i.e., with less-polar comonomer) can control the drug release and the copolymer degradation kinetics. The last work represents a significant approach that may increase the success of the anticancer drugs currently in use.

**Figure 12:**
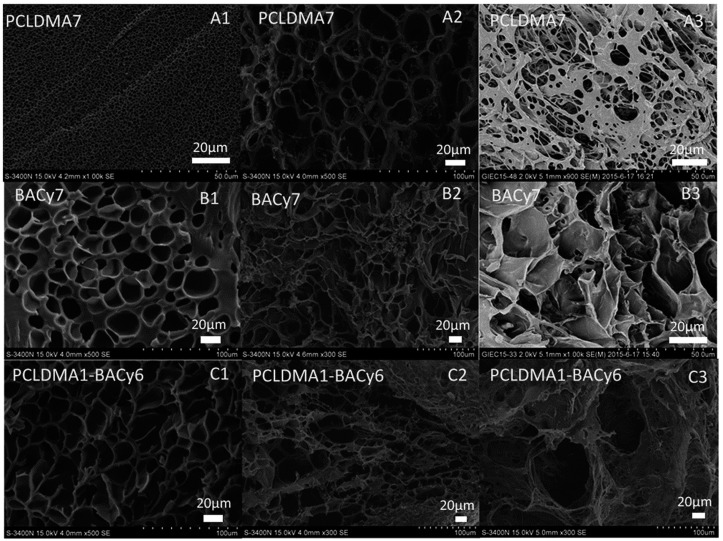
SEM micrographs of degraded (**A1**–**A3**) PNIPAAm-*co*-PCLDMA copolymer, (**B1**–**B3**) PNIPAAm-*co*-BACy, and (**A3**–**C3**) PNIPAAm-*co*-PCLDMA-*co*-BACy at (**A1**–**C1**) 0 days, (**A2**–**C2**) 10 days, and (**A3**–**C3**) 60 days after immersion on glutathione (GSH) at 37 °C. Reprinted with permission from Reference [77]. Copyright © 2016 The Royal Society of Chemistry.

Afterwards, Gan et al. [77] prepared copolymers of NIPAAm with two biodegradable crosslinkers—poly(ε-caprolactone) dimethacrylate (PCLDMA) and the same comonomer employed by Zhan et al. [102], *N*,*N′*-bis(acryloyl)cystamine (BAC)—for the encapsulation and release of the drug levofloxacin (LVF). The synergic combination of PNIPAAm and PCLDMA to enhance the biocompatibility and biodegradability of PNIPAAm thermo-sensitive hydrogels, as well as to obtain thermally controlled monodisperse pore size scaffolds, was previously described by Ratner and co-workers [78]. They noted that the presence of PCL reduces the swelling ratio of PNIPAAm hydrogel, whereas it increases the biodegradability, which in turn was also potentially enhanced due to the incorporation of the comonomer with a disulphide linkage (BAC or BACy, as named in this study). The copolymers were successfully degraded by glutathione (GSH) tripeptide molecules, at 37 °C, over 60 days. Moreover, the cumulative LVF release was very high for PCLDMA4–BACy3, with molar-ratio NIPAAm/PCLDMA/BACy of 700:4:3, in 10 mM of glutathione (GSH) and at 37 °C, reaching almost 100% of cumulative release. SEM micrographs of degraded copolymers (Figure 12) also proved that there is a synergistic effect with the combination of PCL and BAC. The copolymer of PCLDMA1–BACy6 with molar-ratio NIPAM/PCLDMA/BACy of 700:1:6 and the biggest pore size showed high erosion of the hydrogel structure after 60 days of immersion in GSH solution. The aforementioned copolymer had LCST intermediate temperature (31.0 °C) between the pure copolymer PNIPAAm-*co*-PCLDMA7 (30.7 °C) and PNIPAAm-*co*-BACy (32.6 °C).

**Figure 13:**
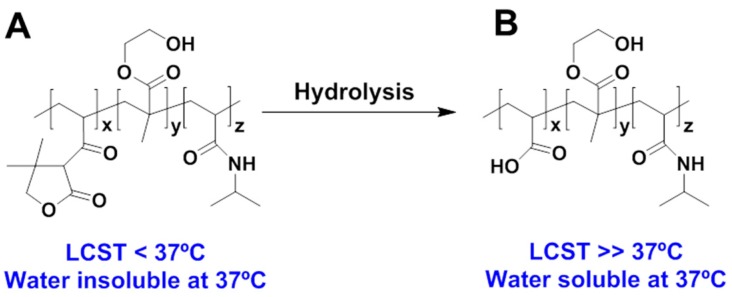
Chemical structures of P(NIPAAm-*co*-HEMA-*co*-DBA) triblock copolymer (**A**) before and (**B**) after hydrolysis of the hydrogel, as reported by Guan and co-workers [75].

Another comonomer which is frequently used with NIPAAm is 2-hydroxyethyl methacrylate (HEMA). This hydrogel is popularly known for its use in contact lens fabrication [103,104]. Guan and co-workers [75] described the preparation of a triblock copolymer composed by NIPAAm, HEMA, and dimethyl-γ-butyrolactone acrylate (DBA), using a PCL derivative as initiator for ATRP copolymerization. The election of DBA was envisaged to enhance the hydrophilicity and degradability of the PNIPAAm thermo-sensitive polymer inside body fluids. This comonomer is able to hydrolyse into acrylic acid (AA). When AA content in hydrolysed PNIPAAm triblock copolymer is 4% or higher, the P(NIPAAm-*co*-HEMA-*co*-AA) possesses a LCST temperature higher than 37 °C (Figure 13), then being stable inside the body for drug delivery studies. On the other hand, the presence of PCL as initiator is supposed to help not only the degradability of the side chains, but also the degradation of the copolymer backbone. The authors tested the in vitro response of their hydrogels, with different stiffness and with or without collagen incorporated, to deliver cardiosphere-derived cells (CDCs) inside myocardial tissue. The CDCs are cell types employed in cardiac cell therapy, and are readily injectable in myocardium to improve cardiac function after hearth attack. On the other hand, the incorporation of collagen is intended to be beneficial to reduce ventricular wall stress commonly observed in infarcted myocardium, thus leading to cardiac function improvement. The outstanding CDC proliferation inside the P(NIPAAm-*co*-HEMA-*co*-DBA) hydrogel combined with the good differentiation of CDC into mature cardiac lineage after a two-week culture period makes this hydrogel a suitable candidate for the delivery of CDCs into infarcted hearts. 

The same research group described most recent papers with chemically modified HEMA comonomers to modulate the LCST, stiffness properties, and CDC encapsulation of PNIPAAm copolymers [105,106].

**Figure 14:**
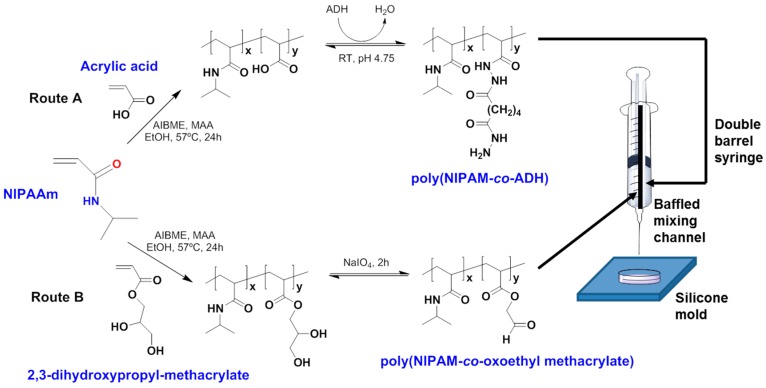
Scheme of the synthesis of hydrazide-functionalized precursor copolymers (PNIPAAm-*co*-ADH) (Route A) and aldehyde-functionalized precursor copolymers (PNIPAAm-*co*-oxoethyl methacrylate) (Route B). ADH is the acronym of adipic acid dihydrazide compound used as reversible and rapid functionalization of PNIPAAm oligomers. Adapted with permission from Reference [76]. Copyright © 2012 American Chemical Society.

**Figure 15:**
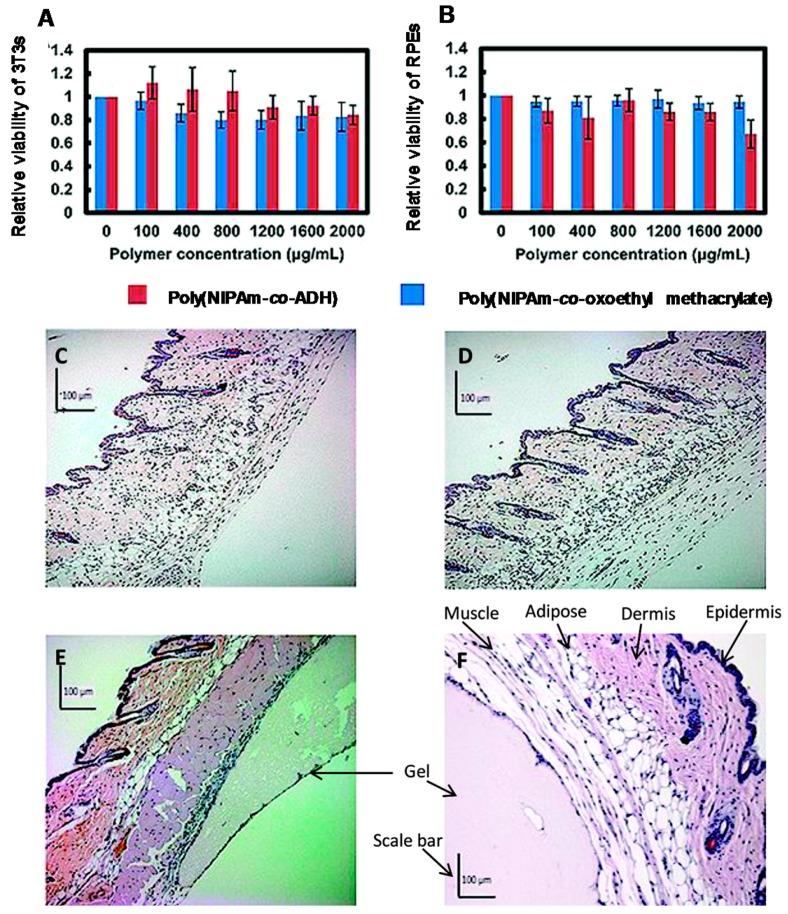
In vitro toxicity assays: (**A**) NIH 3T3 mouse fibroblasts and (**B**) RPE retinal pigment epithelial cells in the presence of (PNIPAAm-*co*-ADH) and (PNIPAAm-*co*-oxoethyl methacrylate). (**C**–**F**) In vivo toxicity assays of the hematoxylin-eosin stained sections of mouse subcutaneous tissue: (**C**) 6 wt % of PNIPAAm-*co*-ADH in PBS, after 48 h; (**D**) 6 wt % of PNIPAAm-*co*-oxoethyl methacrylate in PBS, after 48 h; (**E**) PNIPAAm in situ-formed hydrogel from 6 wt % of polymer precursor solutions in PBS, after 48 h; (**F**) PNIPAAm in situ-formed hydrogel from 6 wt % of polymer precursor solutions in PBS, after 5 months; Tissue labels on (F) are pertinent to all histological samples. Reprinted with permission from Reference [76]. Copyright © 2012 American Chemical Society.

Studies related to in vivo tests for inflammatory accurate analysis after PNIPAAm copolymer implantation has been found. One interesting work is that reported by Patenaude and Hoare [76]. They obtained covalently crosslinked PNIPAAm copolymers with hydrazide and aldehyde functionalities, named poly(NIPAM-*co*-ADH) and poly(NIPAM-*co*-oxoethyl methacrylate), respectively. Both gels—solubilized in NaCl aqueous solutions—were co-extruded to a silicon mold (Figure 14), and a fast network formation (below one minute of reaction) was achieved thanks to the obtaining of hydrazone crosslinked bonds. The co-extruded hydrogel hydrolytically degraded in acid solutions over several weeks into low molecular weight oligomers due to the proton-catalysed rupture of the hydrazone linkages. Additionally, copolymers were used separately for in vitro proliferation assays with NIH 3T3 mouse fibroblasts and retinal pigment epithelial (RPE) cells and in vivo studies by subcutaneous injection on BALB/c mice. The in vitro cell viability assessment showed no cytotoxicity either in NIH 3T3 fibroblasts or in RPE cells (Figure 15A,B). Furthermore, the in vivo toxicity assays demonstrated some inflammatory responses of individual subcutaneous implantation of poly(NIPAM-*co*-ADH) and poly(NIPAM-*co*-oxoethyl methacrylate) hydrogels (Figure 15C,D) 48 h of post-injection, as well as mild inflammatory response for the in-situ crosslinked hydrogel (co-extruded). Surprisingly, no signal of toxicity in adjacent muscle tissues or degradation of the residual hydrogel was found after five months of incubation of the in-situ crosslinked hydrogel (Figure 15E), proving that the hydrazone linkages could play an interesting role for the enhancement of the biocompatibility at longer periods of subcutaneous injection.

**Figure 16:**
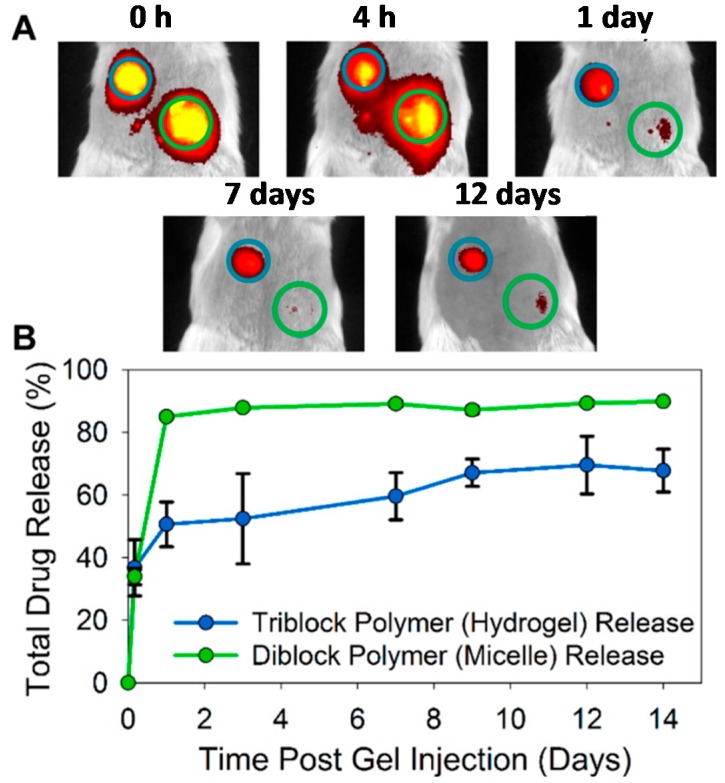
(**A**) Example of in vivo evolution of drug release from diblock copolymer PPS_60_-*b*-PDMA_150_ and triblock copolymer of PPS_60_-*b*-PDMA_150_-*b*-PNIPAAm_150_ injected onto BALB/c mice during 12 days; and (**B**) Quantification of drug release for both hydrogels over 14 days. Reprinted with permission from Reference [22]. Copyright © 2014 American Chemical Society.

As can be seen, there are strong differences in the response of a living organism to the introduction of a diblock copolymer, triblock copolymer, or blended copolymers of PNIPAAm hydrogels. Another fascinating example of the aforementioned differences can be seen in the in vivo study developed by Gupta et al. [22]. They used NIPAAm, hydroxyl functionalized poly(propylenesulfide) (PPS), and *N*,*N*-dimethylacylamide precursors for anionic and RAFT copolymerization. The first is known to act as scavenger for reactive oxygen species by suffering oxidation reactions to poly(propylene sulfoxide) and further poly(propylene sulfone), therefore being useful for gradual drug release and hydrogel degradation. The second is a less hydrophobic monomer than NIPAAm, and is expected to maintain the hydrated state of the hydrogel after in situ gelation. In their work, diblock micelles of PPS_60_-*b*-PDMA_150_ and triblock micelles of PPS_60_-*b*-PDMA_150_-*b*-PNIPAAm_150_ charged with hydrophobic drug (Nile red = dye type) were subcutaneously injected in BALB/c mice. Post-injection, the dye-loaded diblock copolymer micelles diffused away (green circles illustrated on Figure 16A), whereas the dye-loaded triblock copolymer micelles maintained the robustness of the hydrogel (blue circles illustrated on Figure 16A) and the drug released over 14 days (Figure 16B).

Other studies—such as the one developed by Ameer et al. from the Biomedical Engineering Department of Northwestern University (Evanston, IL, USA)—deserve special attention due to the high quality of in vitro and in vivo studies addressed with NIPAAm monomer and biodegradable polymers, such as poly(ethylene glycol)s combined with citric acid (i.e., poly(ethylene glycol citrate)s) [79,80]. They investigated the antioxidant properties of modified PNIPAAm-PEG-citrate hydrogels for the treatment of chronic diabetic foot ulcers, among other applications.

Thermally sensitive polymers with PNIPAAm as main backbones described above are only a few examples showing the importance of a proper selection of the comonomers to potentiate the biodegradability and biocompatibility of the hydrogels for future in vivo implantation.

### 4.2. Poly(N-isopropylacrylamide) Thermo-Responsive Hydrogels with Polyssacharides

PNIPAAm hydrogel has soft architectural structure and relatively high solvent swelling. These characteristics are ideal for living tissue, cells encapsulation, and drug delivery systems, which need soft viscoelastic behaviour to avoid organ damage and a large amount of water to be compatible with living organisms. Nevertheless, its poor mechanical properties (i.e., low elastic modulus, low yield strength and shear stress) are not suitable for the preparation of membranes, surgical meshes, bone repair, or tissue-engineering scaffolds. Therefore, there are several approaches that have been employed in attempts to improve their mechanical properties, including copolymerization, preparation of multi-layered systems, and the development of hybrid materials with natural polysaccharides.

Polysaccharides are attractive materials for potential in vivo applications because of their biocompatibility, biodegradability, and relatively low cytotoxicity, and particularly due to their wide availability from sustainable resources and to their outstanding mechanical properties. Among polysaccharides, cellulose, chitosan, and sodium alginate are some examples which are being explored. Modern methods of polysaccharide isolation and processing have enormously improved their compatibility with other synthetic polymers.

**Figure 17:**
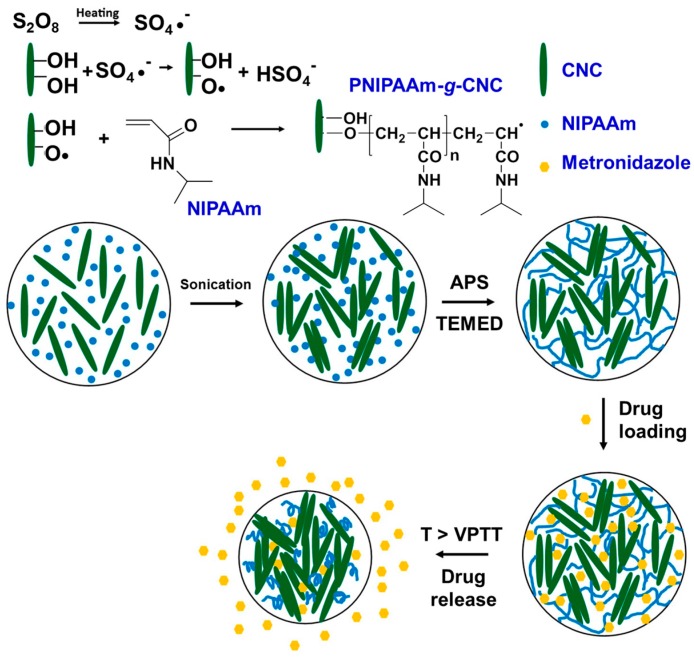
Graft copolymerization of NIPAAm and CNC via free radical polymerization employing ammonium persulfate (APS) as initiator and 1,2-di-(dimethylamino)ethane (TEMED) as accelerator (**up**) and schematic representation of metronidazole drug loading and release upon the action of the volume phase transition temperature (VPTT) at 37 °C (**down**). Adapted with permission from reference [52]. Copyright © 2017 Polymers MDPI.

Zubik et al. [52] grafted NIPAAm onto the surface of cellulose nanocrystals (CNCs) via free-radical polymerization to obtain biocompatible hydrogels without the addition of crosslinkers, for application as wound dressing (i.e., local drug delivery). The synthesis process is represented on Figure 17. Cellulose is a linear syndiotactic homopolymer of β-(1→4)-glycosidic bonds linked to D-anhydroglucopyranose. Cellulose can be found as native cellulose (fibrillar and crystalline) in plants, playing a significant role on the strength of plant cell walls and being ideal as reinforcing material, or can be chemically modified to improve its compatibility with other materials. The idea of employing cellulose nanocrystals with synthetic polymers (such as that reported by Zubik et al.) is due to their excellent elastic modulus comparable to native cellulose (macrocrystals or fibril, about 150 GPa), which is higher than some metals and alloys. However, the major advantage is their significantly high specific surface area compared to native cellulose [107]. The PNIPAAm-*g*-CNC hybrid hydrogels obtained by Zubik et al. [52] exhibited an LCST slightly higher than PNIPAAm homopolymer (from 36 to 39 °C, depending on CNC content) and excellent equilibrium swelling ratio attributed to the hydrophilic groups of CNC. Moreover, the storage modulus (G′) and loss modulus (G″), measured at 37 °C, increased with enhanced content of CNC on grafted PNIPAAm hydrogel. For wound dressing purposes, they encapsulated metronidazole—a synthetic antibiotic effective for the cicatrisation of skin infections. Briefly, in vitro study of metronidazole load and release was carried out with the PNIPAAm-*g*-CNC-50 (highest CNC content grafted with PNIPAAm hydrogel, 50 mg CNC/1 mL of water) over 120 h (pH 7.4, 37 °C). The polymer offered a fast drug release in only 40 min and maximum cumulative drug release (~90%) after only 24 h, which can be beneficial for clinical therapy in hospital infections. In contrast, 10% of metronidazole was retained even after 120 h of test and it is not desirable for a biocompatible material. They ascribed this drug retaining to the high interaction of hydrogen-bonds between the metronidazole molecules and the hydrogel chains, which makes the maximum drug delivery difficult.

Gao et al. [73] used ultraviolet (UV) irradiation to prepare graft copolymers of xylan and PNIPAAm-*co*-AA copolymer. Xylan is the main component of hemicelluloses as well as the major non-cellulosic cell wall polysaccharide, made predominantly of β-d-xylose unit. The resulting xylan-based hydrogel was used to encapsulate acetylsalicylic acid, and further in vitro drug delivery (at pH 1.5 and 7.4) and cell viability (NIH 3T3 cells) studies were carried out. The drug release of the xylan-based PNIPAAm-*co*-AA hydrogels with the maximum drug load efficiency was sensitive to pH, being higher in pH 7.4 than in pH 1.5. They also attributed this behaviour and the remaining 10% of drug to the hydrogen-bond interactions between the acetylsalicylic acid and the xylan chains—the same explanation found for the PNIPAAm and cellulose nanocrystals reported by Zubik et al. On the other hand, excellent biocompatibility with NIH 3T3 cells was achieved after 24 and 72 h of hydrogel incubation.

**Figure 18:**
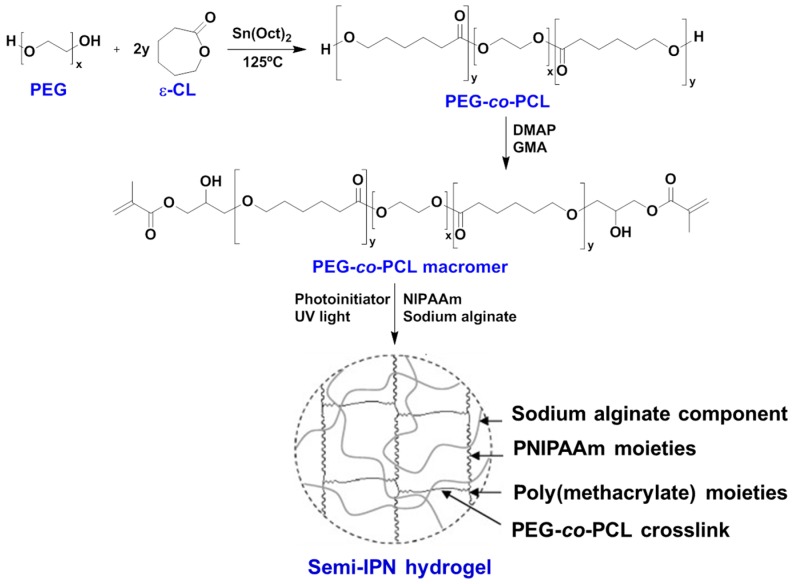
Scheme of the copolymerization reaction between ethylene glycol (EG) and ε-caprolactone (ε-CL) with glycidyl methacrylate (GMA) end-groups and 4-dimethylaminopyridine (DMAP) catalyst, followed by UV irradiation to obtain the interpenetrating polymer network (IPN)-hydrogel with grafted sodium alginate polysaccharide. Adapted with permission from Reference [109]. Copyright © 2009 Elsevier Ltd. (Amsterdam, The Netherlands).

Alginates are a family of water-soluble polysaccharides composed by a block-copolymer structure composed of mannuronic acid and guluronic acid building blocks and mainly used by the food and pharmaceutical industry for specific gelling, thickening, and stabilizing applications. They are capable of phase transition in response to an external temperature stimulus, like PNIPAAm hydrogel. A recent review on the application of alginate biomaterials for regenerative medicine has been published by Sun and Tan [108]. Zhao et al. [109] described the copolymerization of *N*-isopropylacrylamide (NIPAAm) with poly(ethylene glycol)-*co*-poly(ε-caprolactone) (PEG-*co*-PCL) in the presence of sodium alginate to obtain an interpenetrating polymer network (IPN) for drug release studies (Figure 18). The resulting hydrogel structure had improved mechanical strength compared to the hydrogel network without sodium alginate. The storage modulus (G′) and loss modulus (G″) enhanced with increased content of sodium alginate, proving that the polysaccharide was successfully introduced in the hydrogel microstructure. Furthermore, the in vitro bovine serum albumin (BSA) release rate at pH 7.4 and 37 °C was elevated for IPN-hydrogel with the highest concentration of sodium alginate. Thus, their work is a good example how to combine biodegradable synthetic polymers with thermo-responsive hydrogels and biopolymers to obtain more robust hydrogel, improving their biocompatibility and biodegradability.

**Figure 19:**
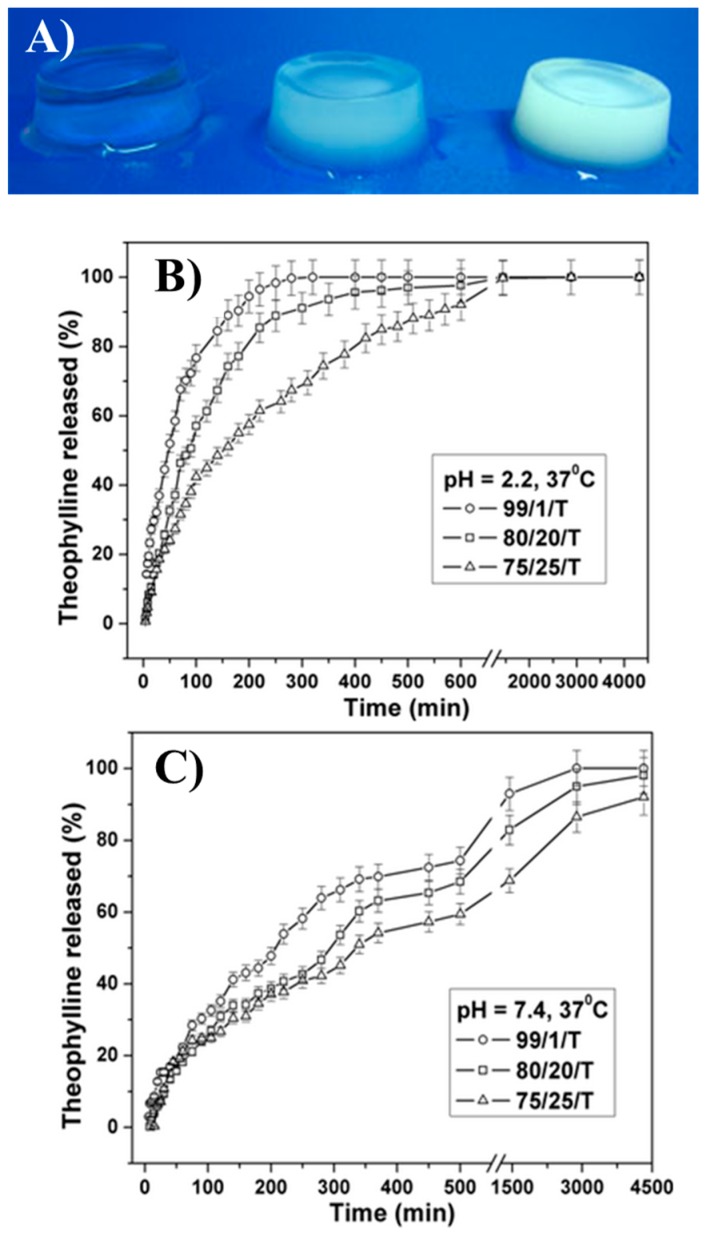
(**A**) Photographs of PNIPAAm-alginate IPN hydrogels; and (**B**,**C**) theophylline release profiles of distinct concentrations of sodium alginate on PNIPAAm hydrogel at pH 2.2 and pH 7.4, respectively. Reprinted with permission from Reference [110] and Reference [74], for (A) and (B,C), respectively. Copyrights © 2010 Society of Chemical Industry and © 2014 Wiley Periodicals Inc. (Seoul, Korea), respectively.

Another good study on the biocompatibility and biodegradability of sodium alginate and PNIPAAm covalently crosslinked with MBA was reported by Vasile and co-workers [74]. The complete synthesis and physical-chemical characterization were described elsewhere [110]. The research team administered the drug theophylline—a potent bronchodilator frequently used on treatment of respiratory diseases—in mice to evaluate the toxicity and biocompatibility of alginate-PNIPAAm IPN hydrogels. Apart from a detailed and new physicochemical study of the characteristics of the theophylline-loaded samples (i.e., their structure, morphology, drug–polymeric matrix interactions, and thermal behaviour), the novelty of this work was the finding of a correlation between drug release and the hydrogel biodegradation, which previously received limited attention. The alginate-PNIPAAm IPN hydrogel (Figure 19A) showed prolonged theophylline release compared to the raw drug injected, 55 h and 24 h, respectively. Moreover, the highest alginate content proved to be more biocompatible than the lowest charged PNIPAAm hydrogel. In vitro theophylline release in acidic media (pH 2.2) was very fast for the lowest concentration of sodium alginate (100% in 5 h, 99/1 NIPAAm to alginate percentage weight ratio) and less burst for the highest concentrations (100% in 24 h, 80/20, and 75/25 NIPAAm-to-alginate percentage weight ratios). The release ratio was dependent on the pH of media, being slower in neutral pH, as can be seen in Figure 19B,C. The system was also able to degrade in the presence of alginate-lyase enzyme—a specific enzyme used particularly for alginate linkage degradation. Degradation of the PNIPAAm/ALG polymeric hydrogels by surface erosion mechanism was revealed, as well as sustained drug release ability.

Other polysaccharides, such as chitosan, guar gum and dextran, have been investigated for obtaining PNIPAAm thermo-sensitive hydrogels [84,85,111,112,113]. Some examples were previously introduced on the biocompatibility and biodegradability section of the present work. However, studies of their in vitro and in vivo assessments are scarce.

### 4.3. Poly(N-isopropylacrylamide) Thermo-Responsive Hydrogels with Polypeptides and Proteins

**Figure 20:**
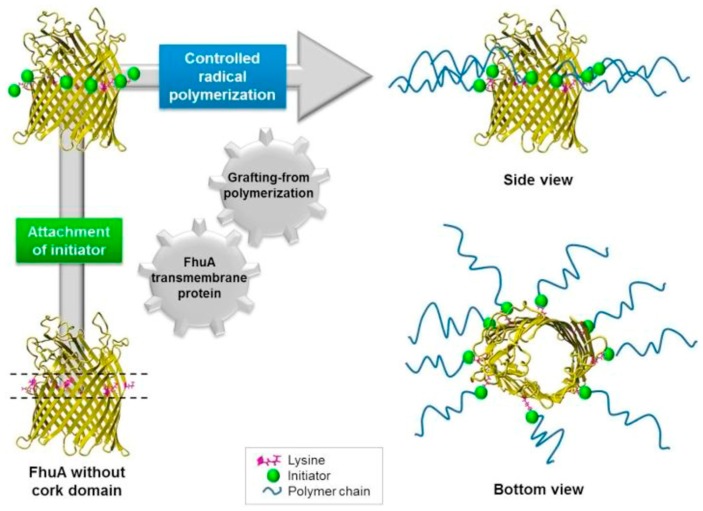
Representation of polymerization of NIPAAm by using β-barrel membrane protein (ferric hydroxamate uptake protein component A, FhuA) with ATRP initiating sites and “grafting from” strategy. Reprinted with permission from Reference [27]. Copyright © 2016 Elsevier Ltd. (Amsterdam, The Netherlands).

PNIPAAm and other thermo-sensitive polymers may also be conjugated to proteins and peptides, making the concept of stimuli-responsive hydrogels particularly appealing for biomedical research. Two methods have been extensively used for the synthesis of protein–PNIPAAm conjugates: (i) “grafting from” method and (ii) “grafting to” method. The first method requires the polymerization of monomers or polymer chains with functional groups directly to a protein, whereas the second needs a prepolymer with reactive end-groups for protein attachment. For example, protein–polymer conjugates were prepared by grafting the thermo-responsive PNIPAAm from the lysine residues of an engineered component of β-barrel membrane proteins—ferric hydroxamate uptake protein component A (FhuA) (Figure 20) [27]. There are several types of β-barrel membrane proteins [114,115], and FhuA is a transmembrane protein isolated from the outer membrane of *Escherichia coli* (*E. coli*). It consists of 22 β-sheets forming a barrel and an *N*-terminal cork domain and particularly possesses remarkable resistance towards high temperature and alkaline pH. Böker and co-workers [27] reengineered the surface residues of FhuA for further controlled radical polymerization of ATRP with initiating sites. The graft polymerization success of FhuA–PNIPAAm conjugates was proved by the reversible water-soluble behaviour at room temperature and hydrophobic state upon raising the temperature above the LCST of PNIPAAm.

Whereas Böker [27] chose to target lysine amino acids to facilitate the growth of polymer chains, Boere et al. [72] preferred cysteine for the preparation of a novel monomer (*N*-(2-hydroxypropyl)methacrylamide-cysteine, HPMA-Cys) to further copolymerize with NIPAAm. l-cysteine is a non-essential sulfur-containing amino acid in humans and an important structural and functional component of proteins and enzymes. They designed a thermo-sensitive triblock copolymer composed of PEG, PNIPAAm, and HPMA-Cys—abbreviated PNC—by very fast in situ covalent crosslinking by native chemical ligation. The key of their strategy was to combine thioester from PEG difunctionalized comonomer or partially functionalized hyaluronic acid (HA) comonomer, and cysteine functionalities. By mixing PNC and PEG or HA solutions at 37 °C, they obtained hydrogels with up to 10 times higher storage modulus (G′) than PNC hydrogel without reaction with thioester groups. The new polymers are envisaged to potentiate the PNIPAAm cell adhesion, cell proliferation, and stem cell differentiation.

**Figure 21:**
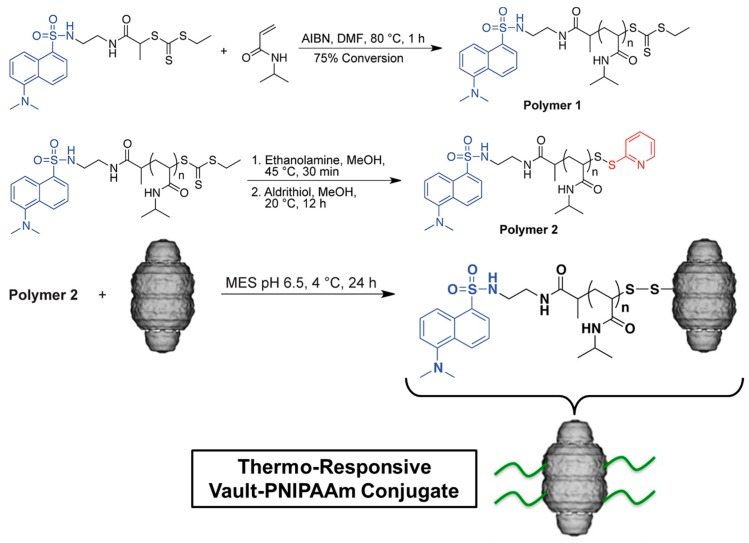
Scheme of the synthesis of thiol-reactive PNIPAAm polymer (Polymer 2) and reversible addition fragmentation chain transfer (RAFT) polymerization with CP-MVP vault. DMF: *N,N*-dimethylformamide; AIBN: azobisisobutyronitrile; MES: 2-(*N*-morpholino) ethanesulfonic acid). Adapted with permission from Reference [118]. Copyright © 2012 American Chemical Society.

Maynard and co-workers have wide experience in the preparation of protein–NIPAAm conjugates by RAFT polymerization [116,117]. Lately, they crosslinked PNIPAAm hydrogel with recombinant vaults obtaining thermo-responsive protein nanocapsules [118]. Vaults are cytoplasmic organelles which are present in many types of eukaryotic cells, and consist primarily of proteins. The protein structure comprises an outer shell composed of 78 copies of the almost 100 KDa major vault protein (MVP). They used cysteine-rich engineered recombinant vaults with thiol-reactive PNIPAAm polymer, as shown in the Figure 21. The LCST of thermo-sensitive hydrogel without vault conjugation was 30.5 °C due to the hydrophobic terminal groups, whereas the cysteine protein (CP)-MVP vault-PNIPAAm conjugates had higher LCST of about 35.9 °C due to the hydrophilic nature of the protein.

Nevertheless, the abovementioned works were centred on the physical-chemical characterization of the novel PNIPAAm bioconjugated polymers, and no degradability or toxicity was investigated. In contrast, some recent reviews have been focused on protein adsorption to PNIPAAm structures, with special emphasis on the mechanisms for controllable cell adhesion and biocompatibility [119,120].

## 5. Novel Applications of PNIPAAm-Based Hydrogel Copolymers and Grafted Polymers

PNIPAAm-based hydrogels are extensively investigated for applications in the controlled delivery of active molecules [121,122], in self-healing materials [45], regenerative medicine [123], tissue engineering [39,124], or in smart encapsulation of cells [125], as mentioned before. However, the widespread commercial availability of end-functionalized PNIPAAm (with a variety of *M*_w_), and PNIPAAm copolymers (with different comonomers) has contributed to the recent explosive growth of innovative materials fabricated with this thermally responsive gel; for example, in obtaining smart surfaces [51,126], nanodevices [127,128], and in vivo 4D-printing systems (also called “4D-bioprinting”) [38,129,130].

PNIPAAm finds widespread application in biointerfaces using hybrid materials and novel technologies. An interesting work was that reported by Zhao et al. [51] (Table 1), who created a cell-inspired biointerface for immunoassays in blood, using copolymers of *N*-isopropylacrylamide (NIPAAm) and sodium acrylate above the surface of a thermoplastic elastomer film composed of styrene-*b*-(ethylene-*co*-butylene)-*b*-styrene (SEBS). The biointerfaces exhibited high resistance to protein and cell adhesion, deformability, and responsiveness dependent on the temperature control—properties relevant for effective in antibody–antigen recognition immunoassay in blood.

The 3D-printing of biocompatible materials and even living cells into 3D functional tissue is a well-known technology [130,131,132]. Due to the incorporation of the fourth dimension (where time is integrated), the printed three-dimensional objects can change their shape or functionalities to other modulated format after an external stimulus is imposed. It is the newest technology recently approached with hydrogels. For instance, a thermo-responsive platform comprising PNIPAAm and a poly(ε-caprolactone) (PCL) bilayer system has been reported. It is able to self-fold and self-unfold in order to encapsulate and deliver cells (yeast cells) in response to temperature changes [38]. The cells were adsorbed on the polymer bilayer at high temperature. Cooling led to hydrogel swelling, and the capsules folded. On the other hand, heating led to capsules unfolding and cells’ release, proving the switching ability of PNIPAAm architectural systems.

Spinks and co-workers, from Intelligent Polymer Research Institute (University of Wollongong), have recently used computer-aided design software (CAD Solidworks) and bioplotter print to fabricate an engineered valve made with alginate/PNIPAAm hydrogel ink [46]. The novelty of their work relies on the design of a method to measure the swelling–deswelling behaviour of the gel by applying a fixed load and by varying the temperature from 20 °C to 60 °C (i.e., below and above the PNIPAAm LCST, respectively). Thanks to the ionic covalently crosslinked alginate-PNIPAAm network (a type of IPN network), they obtained a mechanically robust hydrogel able to support internal and external mechanical loads. Undoubtedly, their technology opens a new insight for the production of hydrogel-based sensors and biomedical devices, such as surgical meshes, cardiovascular stents, or even for the fabrication of bones or cartilage for body implantation.

The use of hydrogel-based biomaterials for biointerface architectures to promote cell adhesion and cell proliferation is of growing interest, as described in the introduction. A nanostructured polymer brush was prepared with PNIPAAm (homopolymer brush) and PNIPAAm-*co*-acrylic acid (binary polymer brush) by ATRP “grafting-to” method [48]. Authors established a correlation between the fibrinogen (FGN) adsorption responsiveness and the hydrophobicity of polymer brushes. Fibrinogen is a blood plasma glycoprotein synthesized in the liver and is particularly interesting for surface adsorption studies. Therefore, higher FNG adhesion was found for binary polymer brushes composed of PNIPAAm and poly(acrylic acid), which also had the highest roughnesses. The amount of FGN protein adsorbed on homopolymer brushes was highly dependent on the analysis temperature. Due to the complex architecture of these systems, many other parameters (e.g., molecular weight, morphology changes, wettability, and cytotoxicity) should be addressed before exploring its potential applications.

**Figure 22:**
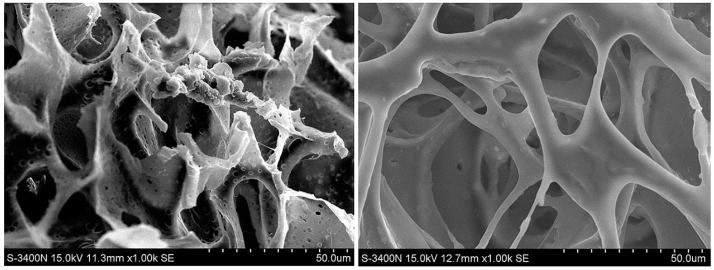
SEM micrographs of PNIPAAm/SWCNT (on **left**) and PNIPAAm hydrogel (on **right**). Reprinted with permission from reference [43]. Copyright from © 2014 Elsevier Ltd. (Amsterdam, The Netherlands).

**Figure 23:**
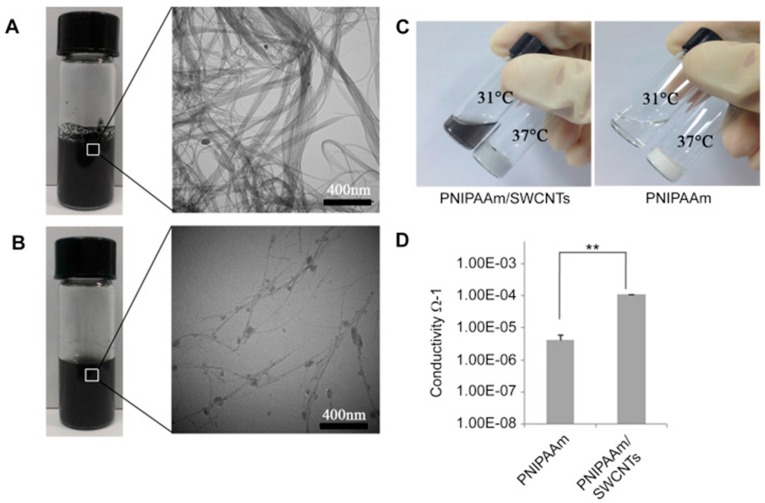
(**A**–**C**) Macroscopic and microscopic images of PNIPAAm and PNIPAAm/SWCNTs hydrogels, (**D**) comparison of conductivities values of both materials, PNIPAAm and PNIPAAm/SWCNTs hydrogels, upon gelation. Reprinted with permission from Reference [43]. Copyright from © 2014 Elsevier Ltd. (Amsterdam, The Netherlands).

A promising hybrid PNIPAAm-carbon nanotube polymer, which is able to carry brown adipose-derived stem cells (BASCs) for treatment of myocardial infarction, was published by Li et al. [43]. The excellent adhesion and survival of BASCs cells on myocardium was attributed to the rough entanglement of the PNIPAAm/single-wall carbon nanotubes (SWCNTs) hydrogels, which favours the longer spreading of BASCs compared to the mostly smooth surface of single PNIPAAm (Figure 22). The SWCNTs incorporated on PNIPAAm hydrogels not only improved the in vivo stem cell transplantation, but also offered conductivity to the system (Figure 23), being useful for the exploration of new biosensors with electrical response.

Some other applications of PNIPAAm hydrogels that are being evaluated include biocidal and anti-fouling surfaces [133] and anti-adhesion or self-cleaning films for optoelectronic and automotive industries [134].

## 6. Brief Conclusions and Future Outlooks

The purpose of this review was to demonstrate that the synthesis of PNIPAAm hydrogel and analogues, as well as the research of new biomaterials employing this stimuli-sensitive gel, is still growing. They combine a unique set of advantages: (i) straightforward and versatile synthesis from commercially available monomers, varying in the nature of end-functionalities and molecular weight; (ii) possible combination of a wide range of materials, including biological compounds, carbon nanotubes, graphene, and ferromagnetic particles; and (iii) extraordinary properties such as thermosensitivity and biocompatibility, and possible modulated biodegradability.

Successful approaches like that reported in the present review may increase the development of PNIPAAm injectable hydrogels for cell delivery and tissue engineering, and as carriers for the supply of anticancer drugs currently in use. It will fruitfully contribute to advances in this biomedical field. Moreover, the high throughput and cost-efficiency of 3D-printing [135,136] will be favourable for researchers to introduce PNIPAAm properties to various substrates and device surfaces. Research works like that published by Spinks and co-workers [46] are only a few examples in this direction.

Furthermore, PNIPAAm-based amphiphilic co-networks systems represent a novel and fascinating class of interpenetrating network, consisting of both hydrophilic and hydrophobic components covalently bonded in macromolecular assembly. Earlier studies, first reported by Lequieu and Du Prez [137], with PNIPAAm segmented polymer networks, showed that in APCNs systems not only the hydrophilic/hydrophobic balance are relevant, but the morphology of the material as well as the crosslink density of the polymer are also determinants for obtaining good permeability response. At present, there exist only a few publications on PNIPAAm-based APCNs, and hence more detailed investigations are needed for this kind of material. Some of the advantages of APCNs for their high impact in the biomedical field are: good biocompatibility, excellent mechanical strength, swelling behaviour independent of solvent polarity, and nanophase separated structure [138].

Unfortunately, in vivo studies are still pending for most PNIPAAm copolymers, grafted polymers, and biopolymer-PNIPAAm conjugates investigated up to today. The major drawbacks are the high cost and ethical restrictions for in vivo analysis to test their viability. Efforts to improve accuracy, reproducibility, and confidence in PNIPAAm hydrogel in vivo assays are highly encouraged.

## Figures and Tables

**Table 1:** Overview of some relevant publications on poly(*N*-isopropylacrylamide) (PNIPAAm) copolymerization and its biomedical applications over the past 10 years.

Authors (Year)	Bioapplications (Bio-Area) *	Scientific Innovation	Improvements in the Biomedical Field
Satarkar et al. (2008) [29]	Remote controlled (RC) drug delivery (D.D.)	High-frequency alternating magnetic field (AMF) to trigger the on-demand pulsatile drug release from nanocomposites synthesized by incorporation of superparamagnetic Fe_3_O_4_ particles in PNIPAAm gels	Application of AMF resulted in uniform heating within the nanocomposites, leading to accelerated collapse and squeezing out large amounts of imbibed drug (release at a faster rate)
Mizutani et al. (2008) [30]	Tissue engineering for endothelial cells (T.E.)	ATRP of PNIPAAm brushes and their influence on the adhesion and the detachment of bovine carotid artery endothelial cells (ECs)	Improvement of surface hydrophilicity, presence of more extended chain conformations with relatively high chain mobility and chain hydration
Klaikherd et al. (2009) [31]	Tuning and control of drug delivery (D.D.)	Novel triple stimuli sensitive block assembly that responds to changes in temperature, pH and redox potential	Fine-tuning of the guest molecule release kinetics and possibility of achieving location-specific delivery
Tan et al. (2009) [32]	Injectable hydrogel for adipose tissue engineering (T.E./S.C.)	Synthesis of copolymer composed by hyaluronic acid and PNIPAAm (AHA-*g*-PNIPAAm)	Encapsulation of human adipose-derived stem cells (ASCs) within hydrogels showed the AHA-*g*-PNIPAAm copolymers were non-cytotoxic and preserved the viability of the entrapped cells
Fujimoto et al. (2009) [33]	Injectable hydrogel for ischemic cardiomyopathy (T.E.)	Biodegradable, thermo-responsive hydrogel based on copolymerization of NIPAAm, acrylic acid (AA) and hydroxyethyl methacrylate-poly(trimethylene carbonate) (HEMAPTMC)	Injection of the material prevented ventricular dilation and improved contractile function in a chronic rat infarction model
Chen et al. (2009) [34]	Blood-compatible materials (T.E.)	Surface-initiated ATRP for PNIPAAm grafting from silicon nanowire arrays	Largely reduced platelet adhesion in vitro, providing a new strategy for fabricating blood-compatible materials
Purushotham et al. (2009) [35]	Anticancer therapy (D.D.)	γ-Fe_2_O_3_ iron oxide magnetic nanoparticles (MNP) coated with PNIPAAm and loaded with anti-cancer drug (doxorubicin-(dox))	Magnetic drug targeting followed by simultaneous hyperthermia and drug release
Yoshida (2010) [36]	Biomimetic actuators (B.S.)	Self-oscillating gels driven by the Belousov-Zhabotinsky reaction	Cyclic soluble–insoluble changes or swelling–deswelling changes without any on–off switching of external stimuli
Wu et al. (2010) [37]	Cancer cell imaging (D.D./B.I.)	Core-shell structured hybrid nanogels composed of Ag nanoparticle (NP) as core and PNIPAAm-*co*-acrylic acid gel as shell	Long circulation and specific accumulation on cells (for use as smart dosing of the pathological zones)
Stoychev et al. (2011) [38]	Yeast cells release (D.D.)	Star-like patterned polycaprolactone-PNIPAAm bilayers like proof of principle for thermo-responsive self-folding capsules	Reversibly encapsulate/release yeast cells in response to temperature signal
Lin et al. (2012) [39]	Cell sheets (S.C.)	Microtextured PNIPAAm-poly(dimethylsiloxane) (PDMS) synthesized by a method suitable for generating aligned vascular smooth muscle cell (VSMC) sheets	Inexpensive, biocompatible, oxygen permeable, and easily microtextured thermo-responsive substrate for producing cell sheets
Dai et al. (2012) [40]	In vivo bioimaging and cancer therapy (D.D./B.I.)	Microspheres of NaYF_4_:Yb^3+/^Er^3+^ coated with PNIPAAm-*co*-(methacrylic acid)] polymer used as carrier for the anticancer drug	Luminescent bioprobes that rapidly release the anticancer drug (doxorubicin hydrochloride, DOX)
Zhu et al. (2012) [41]	Nanogels as microfluidic devices (M.F.D.)	Photothermally sensitive PNIPAAm/graphene oxide (PNIPAAm/GO) nanocomposite synthesized by γ-irradiation	Nanocomposite phase transition is completely reversible via laser exposure or non-exposure
Yang et al. (2013) [42]	Nanocarriers for RC drug release (D.D.)	Near-infrared (NIR)-stimulus controlled drug release system based on Au-nanocage@mSiO_2_@PNIPAAm core–shell nanocarrier	Synergistic chemo-photothermal therapy effect that significantly enhances the cancer cell killing efficacy
Li et al. (2014) [43]	Stem cell transplantation in myocardial repair (S.C.)	A thermo-sensitive single-wall carbon nanotubes (SWCNTs)-modified PNIPAAm hydrogel (PNIPAAm/SWCNTs)	Enhancement of the engraftment of seeding cells in infarct myocardium
Gupta et al. (2014) [22]	Cyto-protective hydrogel for cell encapsulation (D.D.)	ABC triblock polymer poly-[(propylenesulfide)-block-(*N*,*N*-dimethylacrylamide)-block-(PNIPAAm)](PPS-*b*-PDMA-*b*-PNIPAAm)	Good syneresis, lack of degradability, and lack of inherent drug loading and environmentally responsive release mechanisms
Cui et al. (2014) [44]	Injectable hydrogels for cardiac therapy (T.E./S.C.)	Hydrogel composed by PNIPAAm and electroactive tetraaniline (TA) followed by the addition of 2-methylene-1,3-dioxepane (MDO)	2-Methylene-1,3-dioxepane (MDO) and tetraaniline improves biodegradability, electrical properties, and antioxidant activities
Li et al. (2015) [45]	Self-healing hydrogel (T.E.)	Mussel-inspired tri-block copolymer PNIPAAm-*co*-(*N*-3,4-dihydroxyphenethyl acrylamide)]-*b*-poly(ethylene oxide)	Automatic healing from repeated structural damage and effective prevention of non-specific cell attachment and biofilm formation
Bakarich et al. (2015) [46]	Thermally actuating hydrogel for smart valves (T.E./B.S.)	4D Printing of hydrogels made by interpenetrating network of alginate and PNIPAAm	Mechanically robust and thermally actuating 4D printed smart valve
Kesti et al. (2015) [47]	Bioink for articular cartilage (T.E.)	Blending of PNIPAAm grafted hyaluronan (HA-PNIPAAm) with methacrylated hyaluronan (HAMA)	High-resolution scaffolds with good viability printed layer-by-layer
Psarra et al. (2015) [48]	Protein adsorption and cell adhesion (T.E.)	Nanostructures of PNIPAAm (homo) and PNIPAAm-*co*-acrylic acid (binary) by atom transfer radical polymerization (ATRP) and investigation of the fibrinogen (FGN) adsorption responsiveness	Terminal hydrophobic moieties improved wettability, lower critical solution temperature (LCST), and morphology of both brush systems with consequent alteration of FGN adsorption
Lima et al. (2016) [49]	Ocular biocompatibility (T.E.)	Study of the safety of intravitreal injections of poly-*N*-isopropylacrylamide (PNIPAAm) tissue adhesive in rabbit eyes	Intravitreal injections of PNIPAAm were nontoxic in this animal study
Li et al. (2016) [50]	Stem-cell carriers for cardiac therapy (S.C.)	Free-radical polymerization of NIPAAm, propylacrylic acid, hydroxyethyl methacrylate-*co*-oligo(trimethylene carbonate), and methacrylate poly(ethylene oxide) methoxy ester	Innovative hydrogels that quickly solidify at the pH of an infarcted heart but cannot solidify at the pH of blood injectable through catheters, commonly used for minimally invasive surgeries
Zhao et al. (2017) [51]	Cell-inspired biointerface for use in immunoassays in blood (T.E./B.S.)	Biointerfaces constructed by patterning smart hydrogels poly(*N*-isopropylacrylamide-*co*-sodium acrylate) (PNIPAAm-*co*-PNaAc) on hydrophilic layers (poly(ethylene glycol), PEG), followed by immobilization of antibodies on the patterned hydrogels	Versatile and effective biointerfaces for antibody–antigen recognition, which offers a potential new approach for developing highly sensitive immunoassays in blood
Zubik et al. (2017) [52]	Wound dressing (T.E.)	PNIPAAm reinforced with cellulose nanocrystals (CNCs); for wound dressing purposes, metronidazole was used as a target drug	Injectable hydrogels as promising materials for wound dressing
Liu et al. (2018) [53]	Photosensitizer for cancer treatment (D.D.)	A novel comb-shaped porphyrin end-functionalized poly(NIPAAm)-*b*-poly[oligo (ethylene glycol methyl ether methacrylate)]	Photo-toxicity toward HeLa cancer cells and local accumulation on tumor tissues: photosensitizer in photodynamic anticancer therapy

* Notes: Abbreviations for the bio-area of PNIPAAm studies: Drug Delivery (D.D.); Tissue Engineering (T.E.); Bio-Sensor (B.S.); Bio-Imaging (B.I.); Microfluidic Devices (M.F.D.).
